# Mechanistic Insights into the Seed-Mediated Growth of Perovskite Nanostructures via a Two-Step Dissolution–Recrystallization Method

**DOI:** 10.3390/ma18122858

**Published:** 2025-06-17

**Authors:** Se-Yun Kim

**Affiliations:** Department of Advanced Materials Science and Engineering, Kyungnam University, Changwon-si 51767, Republic of Korea; kimseyun@kyungnam.ac.kr

**Keywords:** organo-metal halide perovskite, two-step process, nanostructure, formation mechanism of perovskite nanostructure, Ostwald ripening

## Abstract

In this study, we investigated the formation mechanism of organo-metal halide perovskite nanostructures through a two-step process categorized as dissolution–recrystallization. It is proposed that the initial formation of nanostructures is governed by the generation of seed grains, whereas the Ostwald ripening model explains only the subsequent growth stage of these structures. We suggest that newly generated grains—formed adjacent to pre-positioned grains—experience compressive stress arising from volume expansion during the phase transition from PbI_2_ to the MAPbI_3_ perovskite phase. Owing to their unstable state, these grains may serve as effective seeds for the nucleation and growth of nanostructures. Depending on the dipping time, diverse morphologies such as nanorods, plates, and cuboids were observed. The morphology, including the aspect ratio and growth direction of these nanostructures, appears to be strongly influenced by the residual compressive stress within the seed grains. These findings suggest that the shape and aspect ratio of perovskite nanostructures can be tailored by carefully regulating nucleation, dissolution, and growth dynamics during the two-step process.

## 1. Introduction

Recently, lasing from single-crystal organo-metal halide perovskite nanowires with exceptionally low lasing thresholds and high Q-factors has been reported [1]. The outstanding lasing performance of organolead halide perovskite nanowires can be attributed to their long carrier lifetimes and low non-radiative recombination rates. Given their unique rectangular nanowire geometry, tunable emission across the visible spectrum via mixed halides, ease of single-crystal perovskite and nanostructure growth in solution phase at room temperature, and superior lasing characteristics, lead halide perovskites—including cesium lead halide perovskites—are considered promising candidate materials for a wide range of applications, such as nanophotonics and chemical/biological sensing [1,2].

Understanding the growth mechanisms of perovskite nanostructures is essential for controlling their aspect ratios and diameters, which are critical parameters for lasing applications. For example, in the case of CsPbBr_3_ nanowires, diameters greater than 180 nm are required to achieve lasing, as ultrathin nanowires (~20 nm in diameter) cannot support photonic lasing modes [1,3]. Although many studies have reported the promising lasing properties of perovskite nanostructures, a comprehensive and mechanistically sound explanation of their growth—particularly via the two-step process—has not yet been fully established [1,2,4].

In general, the formation of nanostructures with high aspect ratios in solution is typically achieved through the oriented attachment of nanocrystals, screw dislocation-driven growth, or surfactant-assisted growth. In oriented attachment, nanoparticles—often only a few nanometers in size—can continuously rotate until they find an aligned crystallographic orientation, after which they irreversibly attach to each other via lateral atom-by-atom addition at the interface [5,6]. In screw dislocation-driven growth, axial screw dislocations provide a self-perpetuating step that propagates in a spiral trajectory around the Burgers vector of the dislocation [7]. In surfactant-assisted growth, the adsorbed species, such as surfactant molecules, inorganic ions, or small molecules, can selectively slow down the crystal growth of specific facets where they are adsorbed, while promoting the growth of other facets [8].

In the case of organo-metal halide perovskites, it has been suggested that perovskite materials with intrinsic polarization may follow an oriented attachment mechanism during nanocrystal growth. However, Dandan Zhang et al. reported that the formation of colloidal CsPbX_3_ nanowires is unlikely to proceed via dipole-driven one-dimensional oriented attachment [3]. Haiming Zhu et al. proposed that nanowire growth occurs via a dislocation-driven crystal growth mechanism, in which initial dislocation defects present on the surface of polycrystalline CH_3_NH_3_PbI_3_ films trigger the nucleation of perovskite nanowires [1]. Nevertheless, the dislocation-driven model cannot fully explain the formation of various nanostructure morphologies under identical growth conditions, nor the specific conditions required for controlled growth.

In this study, we propose a growth mechanism for perovskite nanostructures based on the Ostwald ripening model and offer key insights into controlling their aspect ratios. Furthermore, we investigated the origin of morphological diversity in perovskite nanostructures, including nanorods, plates, and cuboids.

## 2. Materials and Methods

CH_3_NH_3_I was synthesized by reacting 27.8 mL of CH_3_NH_2_ (40 wt% in methanol, Tokyo Chemical Industry Co., Ltd., Tokyo, Japan) with 30 mL of HI (57 wt% in water, Sigma-Aldrich, Inc. St. Louis, MO, USA) in a round-bottom flask under continuous stirring for 2 h in an ice bath. The resulting CH_3_NH_3_I was collected using a rotary evaporator at 50 °C for 1 h, washed several times with diethyl ether, and dried under vacuum for 24 h. PbI_2_ solutions were prepared by dissolving 462 mg of PbI_2_ (99%, Sigma-Aldrich, Inc., St. Louis, MO, USA) in 1 mL of N,N-dimethylformamide (DMF, 99.8%, Sigma-Aldrich, Inc., St. Louis, MO, USA) at 70 °C to obtain a 1 M solution. A 30 μL aliquot of the PbI_2_ solution was spin-coated onto a glass substrate at 3000 rpm for 30 s, followed by drying at 100 °C for 5 min. Two sets of experiments were conducted to investigate the growth behavior of perovskite nanostructures:

(1) The PbI_2_-coated films were immersed in 5 mL of MAI/IPA solution at various concentrations—0.038 M (6 mg/mL) and 0.063 M (10 mg/mL)—for 20 s, 2 min, 10 min, 30 min, and 60 min at room temperature.
(2) The PbI_2_ film treated with 0.315 M (50 mg/mL) solution for 5 s and the untreated PbI_2_ film were both subsequently immersed in 0.038 M solution for 12 h at room temperature.

The morphologies and microstructures of the resulting films were characterized using field-emission scanning electron microscopy (FE-SEM, Hitachi, SU8020, Tokyo, Japan). The crystal structure of the perovskite films was analyzed using high-resolution X-ray diffraction (HR-XRD, PANalytical, Almero, The Netherland) with Cu Kα radiation under operating conditions of 40 kV and 30 mA.

## 3. Results and Discussion

**Figure 1:**
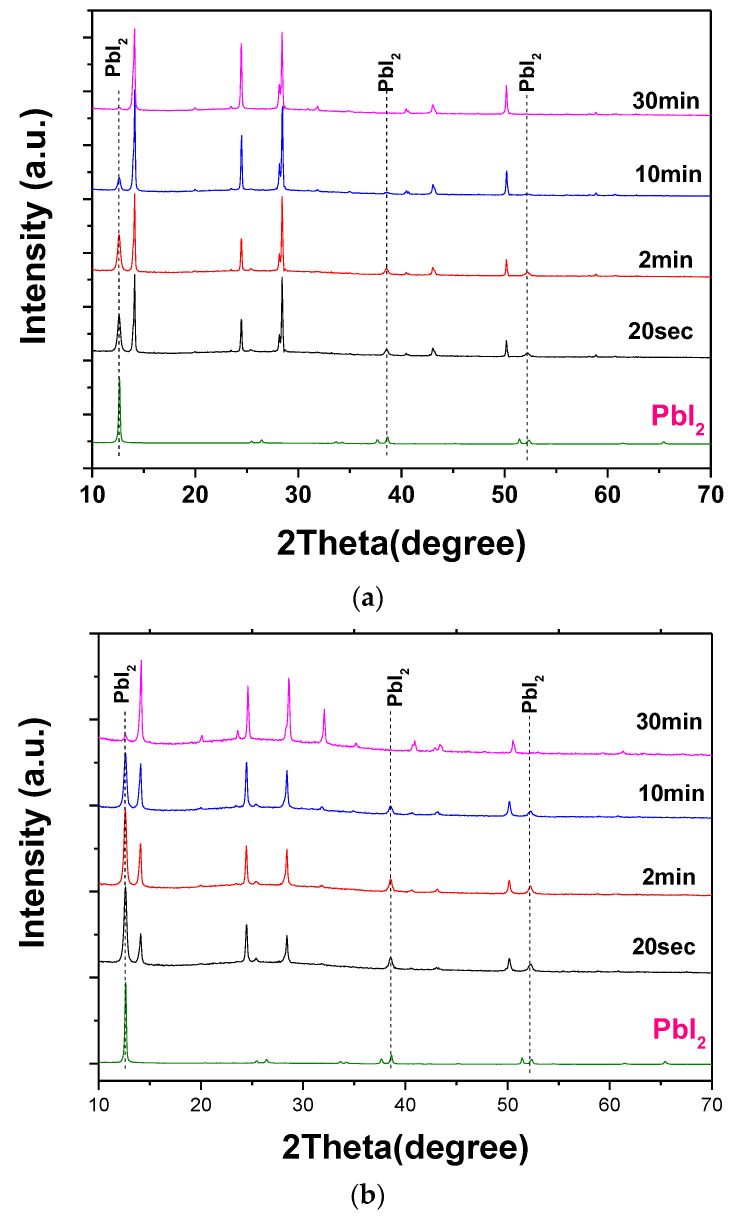
XRD patterns of perovskite films prepared with different dipping times in MAI solutions of (**a**) 0.038 M and (**b**) 0.063 M.

**Figure 2:**
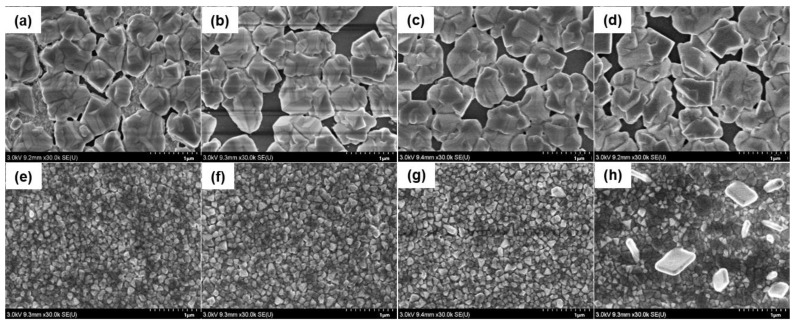
FE-SEM images of perovskite films prepared using (**a**–**d**) 0.038 M MAI solution for (**a**) 20 s, (**b**) 2 min, (**c**) 10 min, and (**d**) 30 min, and (**e**–**h**) 0.063 M MAI solution for (**e**) 20 s, (**f**) 2 min, (**g**) 10 min, and (**h**) 30 min.

Figure 1a,b show the XRD patterns of perovskite films prepared by dipping PbI_2_-coated substrates in 0.038 M and 0.063 M MAI solutions for various durations, respectively. The diffraction peak at 12.6° observed in the PbI_2_ films corresponds well to the (001) lattice plane of the hexagonal 2H PbI_2_ polytype (JCPDS No. 07-0235). The diffraction peaks of the resulting perovskite films match well with those of the tetragonal perovskite phase [9]. As shown in Figure 2a,b, the PbI_2_ peaks gradually decrease with increasing dipping time under both concentration conditions. For the 0.038 M solution, the residual PbI_2_ rapidly disappeared, indicating efficient conversion. In contrast, when using the 0.063 M solution, a substantial amount of unconverted PbI_2_ remained, even after 10 min of dipping. These results indicate that the conversion rate of PbI_2_ to perovskite differs significantly between the two concentrations, particularly during the initial stages of the reaction.

Figure 2 shows FE-SEM images of perovskite films formed at two different MAI concentrations—0.038 M and 0.063 M—after various dipping durations: 20, 120, 600, and 1800 s. Figure 2a–d correspond to films prepared using the 0.038 M MAI solution for 20 s, 2 min, 10 min, and 30 min, respectively. In this case, isolated large perovskite grains with faceted surfaces were observed, but no nanorod or plate-like nanostructures appeared, even with prolonged dipping. The morphology remained dominated by individual faceted grains.

In contrast, the use of the 0.063 M MAI solution resulted in the formation of continuous perovskite films composed of smaller grains, as shown in Figure 2e–h. Notably, early-stage nanostructure seeds were observed after 600 s of dipping, as shown in Figure 2g and Figure S1. Fully developed nanostructures, including nanorods, plates, and cuboids, were clearly identified after 1800 s of dipping in the 0.063 M solution, as shown in Figure 2h. Haiming Zhu et al. proposed a dislocation-driven mechanism for the formation of perovskite nanowires [1]. If such nanostructures were indeed formed via dislocation sites at grain boundaries, similar nanostructures would be expected to appear under both MAI concentrations. However, as seen in Figure 2a–d, no nanostructures were observed in the films prepared using the 0.038 M solution. Furthermore, as shown in Figure S2, nanostructures, such as rods, plates, and cuboids, did not originate from localized defects within the grains; rather, the grains themselves appeared to act as seeds for growth. These results suggest that the nanostructures formed via the two-step process are not the result of a dislocation-driven growth mechanism.

To understand the formation mechanism of nanostructures, it is essential to consider the Ostwald ripening model, which describes the coarsening of particles in a liquid medium through material transport from smaller particles to larger ones. The relationship between chemical potential and particle radius (or surface energy) demonstrates that smaller particles (or those with higher surface energy) are always energetically less stable than larger ones (or those with lower surface energy). In other words, the concentration of dissolved components near smaller particles is higher than that near larger particles. This concentration gradient drives mass transport from small grains to large grains, resulting in size-selective growth.

In the two-step perovskite formation process, the phase transition from PbI_2_ to the perovskite phase occurs when dried PbI_2_ films are dipped into the MAI (CH_3_NH_3_I) solution in isopropanol (IPA). Perovskite nuclei are formed as a result of a spontaneous chemical reaction, as shown in Equation (1) [4,10,11,12]. Subsequently, the dissolution and growth processes of perovskite structures follow the mechanisms described in Equations (2) and (3).

(1) $$\mathrm{Nucleation}:\;PbI_{2}(solid)+MAI(solution)\to MAPbI_{3}(solid)$$

(2) $$\mathrm{Dissolution}:MAPbI_{3}(solid)\to Dissolved\;components(solution)$$

(3) $$\mathrm{Growth}:MAPbI_{3}\left(solid\right)\leftarrow Dissolved\;components(solution)$$

The spontaneous chemical reaction described in Equation (1) is considered critical for creating a local environment conducive to seed formation. The perovskite nuclei initially formed can dissolve in the IPA solution, as shown in Equation (2), and their subsequent growth can proceed via the re-precipitation of dissolved species, as described in Equation (3).

**Figure 3:**
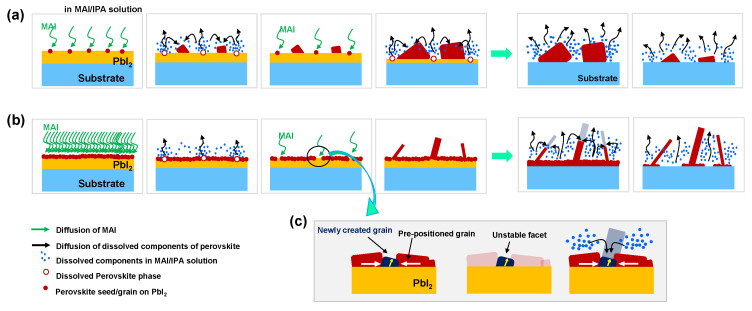
Schematic diagram of perovskite growth behavior for a PbI_2_-coated sample in (**a**) a low-concentration MAI solution and (**b**) a high-concentration MAI solution. (**c**) Seed and nanostructure formation behavior generated by dissolution and re-formation in a high-concentration MAI solution.

Figure 3 illustrates the proposed formation mechanism of perovskite nanostructures via the Ostwald ripening process. When a low-concentration MAI solution is used, a low density of initial perovskite nuclei forms on the PbI_2_ layer, as depicted in Figure 3a. Through Ostwald ripening, smaller particles shrink while larger ones grow, driven by differences in chemical potential. The source of the dissolved components in IPA is the dissolution of pre-existing perovskite grains. Grain growth continues as long as a concentration gradient exists between the grain surface and the surrounding solution. Under these conditions, perovskite grains may eventually dissolve completely with prolonged immersion in IPA, since the local concentration gradient induces continuous outward diffusion from the grains into the solvent. Consequently, nanostructures cannot form in this scenario.

However, a high density of initial perovskite nuclei on PbI_2_ forms when a high-concentration MAI solution is used, as illustrated in Figure 3b. After a dense perovskite layer forms on the PbI_2_ surface via the spontaneous reaction described in Equation (1), further phase transformation from PbI_2_ to perovskite becomes limited due to restricted MAI diffusion through the pre-existing perovskite layer. Additional phase conversion can only occur at the exposed PbI_2_ surface through the dissolution of pre-positioned perovskite grains, as described in Equation (2). At this stage, it is proposed that seeds for perovskite nanostructure growth can form.

Newly generated grains at the exposed PbI_2_ surface are formed via Equation (1) and serve as seeds for nanostructure growth, as illustrated in Figure 3c. It is hypothesized that these newly formed grains experience compressive stress due to volume expansion during the phase transition within the confined space surrounded by pre-positioned grains. Notably, the densities of PbI_2_ and MAPbI_3_ are 6.16 and 4.22 g/cm^3^, respectively. The compressive stress of the grain is expected to be partially relieved through structural distortion toward an open space—typically the upward direction—if the grain is encapsulated by surrounding perovskite.

The cross-sectional dimension of the resulting rod-like nanostructure may depend on the size of the initial seed, whereas the growth direction may be influenced by the direction of stress-induced distortion. For instance, the aspect ratios and cross-sectional sizes of the nanorods vary significantly, as shown in Figure S3. These newly created grains may possess unstable (e.g., polarized) facets induced by distortion, which preferentially attract dissolved species originating from the surrounding pre-positioned grains. Ultimately, nanostructure growth proceeds through this preferential deposition on unstable facets.

For these reasons, it is proposed that perovskite nanostructures form under the 0.063 M MAI condition. In contrast, compressive stress is absent in the films formed using the 0.038 M MAI solution, which explains the presence of only large, isolated grains without nanostructure formation, as observed in Figure 2a–d.

To investigate the effect of MAI concentration on seed density and the mass transport of dissolved components from perovskite grains to seeds, both non-treated and pre-treated PbI_2_ films were immersed in 0.038 M MAI solution for 12 h. The pre-treated PbI_2_ films were prepared by dipping them in an approximately 0.3 M MAI solution for 5 s.

**Figure 4:**
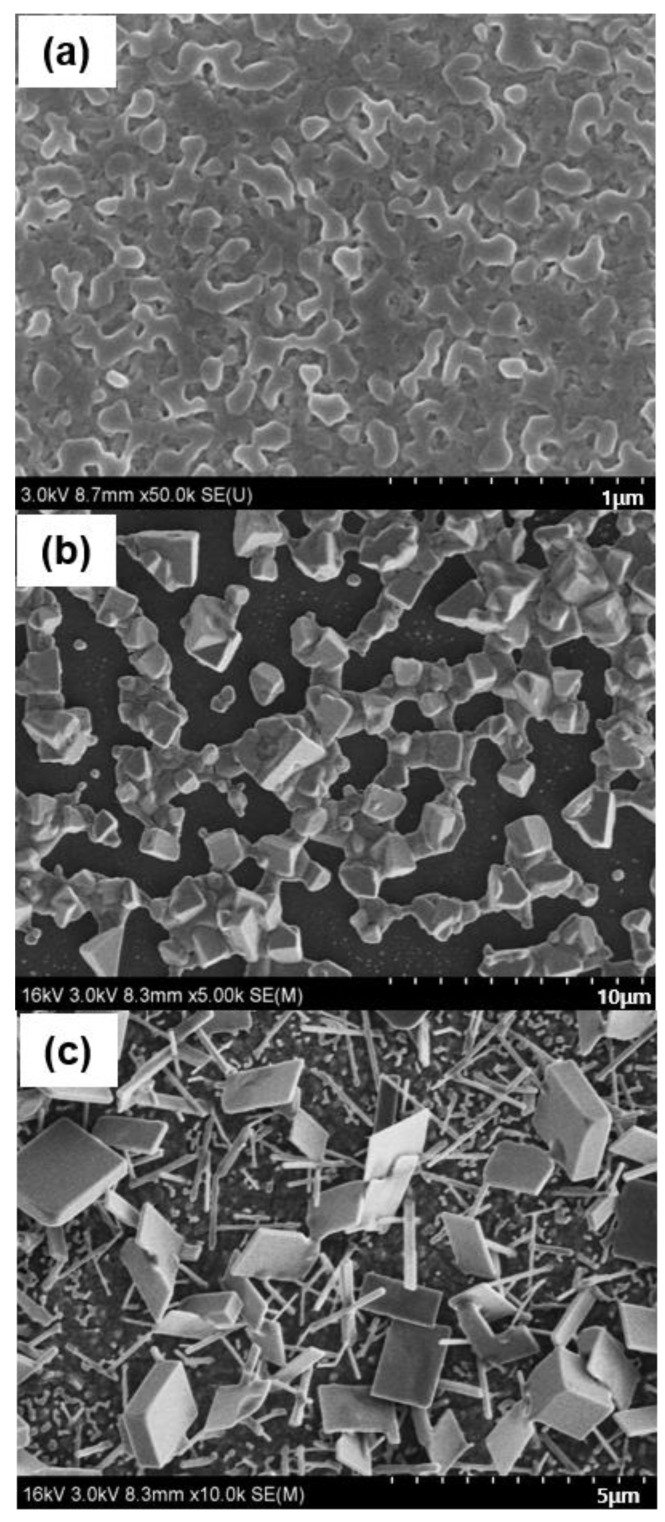
FE-SEM images of (**a**) the as-deposited PbI_2_ film, (**b**) the perovskite film formed from a non-treated PbI_2_ film, and (**c**) the perovskite nanostructure formed from a treated PbI_2_ film. The treated PbI_2_ film was prepared by dipping it in a 0.315 M (50 mg/mL) MAI solution for 5 s. Both films were subsequently immersed in a 0.038 M MAI solution for 12 h.

Figure 4a–c show FE-SEM images of the as-deposited PbI_2_ film, the perovskite film formed from the non-treated PbI_2_, and the film formed from the treated PbI_2_, respectively. In Figure 4b, isolated small perovskite grains were observed without any nanostructures. In contrast, Figure 4c reveals the formation of nanostructures, such as rods, plates, and cuboids.

In both cases, exposed substrates were observed due to the dissolution of perovskite in the IPA solvent. It was found that the size of perovskite grains in the films prepared from untreated PbI_2_ gradually decreased with increasing dipping time. In contrast, nanostructures persisted in the films prepared from pre-treated PbI_2_ under the same dipping conditions, although the nanostructures exhibited slight curvature.

The following conclusions can be drawn from Figure 2, Figure 3 and Figure 4:

1. The density of perovskite seeds is dependent on the MAI concentration. Also, it is anticipated that the porosity (i.e., specific surface area) and crystallographic orientation of PbI_2_ play a critical role in determining the initial nucleation density. Hence, further systematic investigations are required to clarify their exact influence.
2. The Ostwald ripening growth model is applicable to the two-step perovskite formation process.
3. Thermodynamically unstable phases can serve as seeds for nanostructure formation, with morphological evolution occurring toward thermodynamically stable phases.
4. Nanostructure growth depends on the existence of seeds, whereas the Ostwald ripening model alone describes only grain coarsening.
5. Newly formed grains surrounded by pre-positioned perovskite grains may experience compressive stress due to volume expansion during the phase transition from PbI_2_ to the perovskite phase, and these grains can act as seeds for nanostructure growth due to their unstable state.

**Figure 5:**
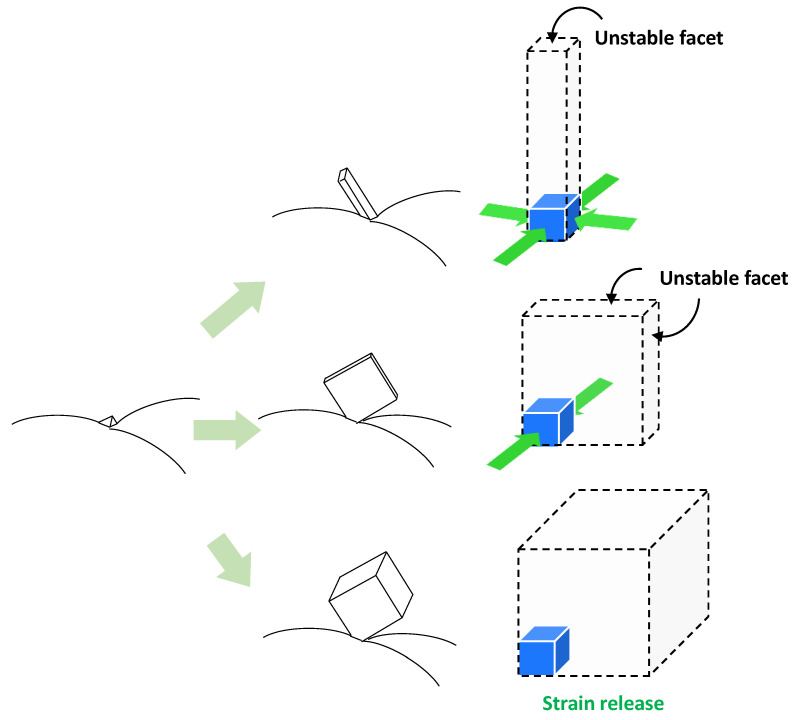
Schematic illustration of the formation mechanism of perovskite nanorod, plate, and cuboid structures.

Figure 5 presents a schematic illustration of the morphological evolution of perovskite nanorods, plates, and cuboids. As previously discussed, structural distortion in newly created grains with unstable facets can be induced by the surrounding pre-positioned grains. It is proposed that rod-shaped perovskite nanostructures result from bi-directional compressive stress, whereas plate-shaped nanostructures are formed under unidirectional compressive stress.

The facet not associated with compressive stress is likely a thermodynamically unstable facet, to which dissolved perovskite components can attach. In the case of cuboid structure formation, it is estimated that compressive stress within the seed plays a role in the initial nucleation step. However, this stress is gradually relieved through the dissolution of surrounding pre-positioned grains that induced the stress, ultimately leading to the formation of cuboid perovskite structures via partial stress release.

Interestingly, in addition to nanorod, plate, and cuboid structures, transitional morphologies—ranging from rod to plate, or plate to cuboid—were also observed, as shown in Figure S4. This suggests that compressive stress within the seed can be gradually released as dipping time increases. In other words, the shape of the resulting nanostructure can be modulated by controlling compressive stress.

If nanostructure shape is governed by the relaxation of compressive stress due to the dissolution of pre-positioned grains, then the key reaction for controlling both shape and aspect ratio is the dissolution process of these grains. Consequently, factors such as the choice of solvent, its temperature, and MAI concentration are critical in tuning nanostructure morphology and dimensions. Ongoing research is focused on optimizing nanostructure morphology by precisely regulating the dissolution behavior, as described in Equation (2).

The following conclusions and hypotheses are proposed:

1. The dissolution reaction is temperature dependent. At lower temperatures, slower dissolution leads to fewer and thinner nanostructures, whereas higher temperatures promote faster dissolution and result in a greater density of thicker structures.
2. The growth rate of nanostructures depends on the concentration of dissolved perovskite components. The aspect ratio of the resulting structures can be modulated by tuning this concentration.
3. A sequential process may enable control over both thickness and aspect ratio. Seed density and type can be governed by solvent temperature, whereas nanostructure growth rate is regulated by the concentration of dissolved perovskite species.

Although the shape, aspect ratio, and spatial distribution of perovskite nanostructures could not be fully controlled in this study, experimental evidence supporting morphology evolution mechanisms has been successfully obtained.

## 4. Conclusions

In this study, we proposed a formation mechanism for perovskite nanostructures via a two-step process, which can be categorized as a dissolution–recrystallization pathway. The density of perovskite seeds was found to depend on the MAI concentration. The Ostwald ripening model was applicable to the nanostructure growth observed in this two-step process, and the presence of seeds was essential for the formation of nanostructures.

We further suggested a seed formation mechanism in which newly created grains, surrounded by pre-positioned grains, experience compressive stress due to volume expansion during the phase transition from PbI_2_ to the perovskite phase. These grains, being in a thermodynamically unstable state, can act as seeds for nanostructure growth. Various nanostructure morphologies—including rods, plates, and cuboids—were observed, with variations in aspect ratio and growth direction. These morphological differences are assumed to arise from differences in the degree and direction of remaining compressive stress within the seed grains.

As a future direction, we suggest that the shape and aspect ratio of perovskite nanostructures can be effectively controlled by regulating the dissolution dynamics of pre-positioned grains.

## Data Availability

The original contributions presented in this study are included in the article/Supplementary Material. Further inquiries can be directed to the corresponding author.

## Supplementary Materials

The following supporting information can be downloaded at: https://www.mdpi.com/article/10.3390/ma18122858/s1, Figure S1: FE-SEM image showing various seed morphologies for perovskite nanostructures formed after 600 s of dipping in a 0.063 M MAI solution; Figure S2: FE-SEM image showing the cross-sectional view of a perovskite nanorod; Figure S3: FE-SEM images showing the dimensional variation and aspect ratios of perovskite nanorods; Figure S4: FE-SEM images of nanorods, plates, and cuboids exhibiting unique transitional shapes, such as rod-to-plate and plate-to-cuboid transformations.
