# Supplementary material for: Mechanistic Insights into the Seed-Mediated Growth of Perovskite Nanostructures via a Two-Step Dissolution–Recrystallization Method
Source: Materials (Basel). 2025 Jun 17;18(12):2858. doi: 10.3390/ma18122858 (PMC12195002; doi:10.3390/ma18122858)
Supplement: Supplementary file 1 [file materials-18-02858-s001.zip › materials-3660054-supplementary.pdf]

Supporting information

Mechanistic Insights into the Seed-Mediated Growth of Perovskite Nanostructures via a Two-Step Dissolution–Recrystallization Method

Se-Yun Kim

Department of Advanced Materials Science and Engineering, Kyungnam University, Changwon 51767, Republic of Korea

*Corresponding author Email: kimseyun@kyungnam.ac.kr

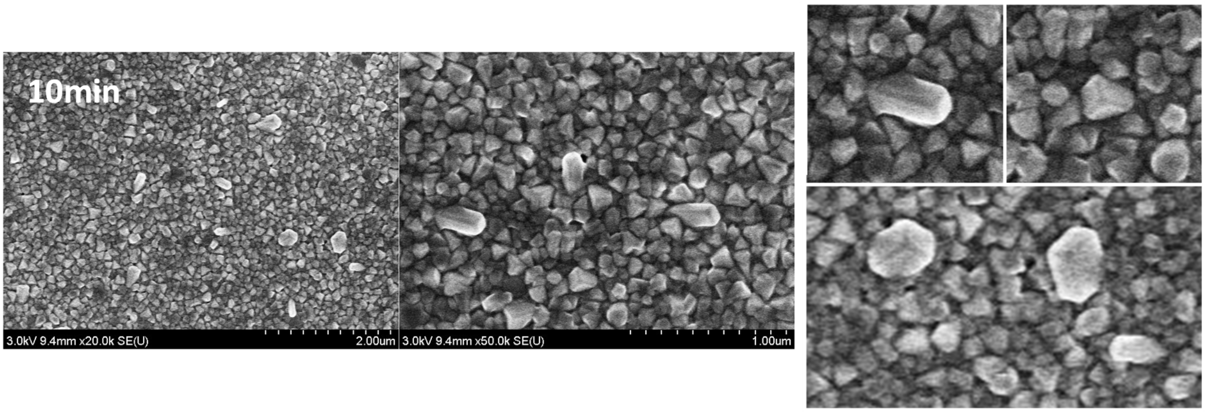

Figure S1. FE-SEM image showing various seed morphologies for perovskite nanostructures formed after 600 seconds of dipping in 0.063 M MAI solution.

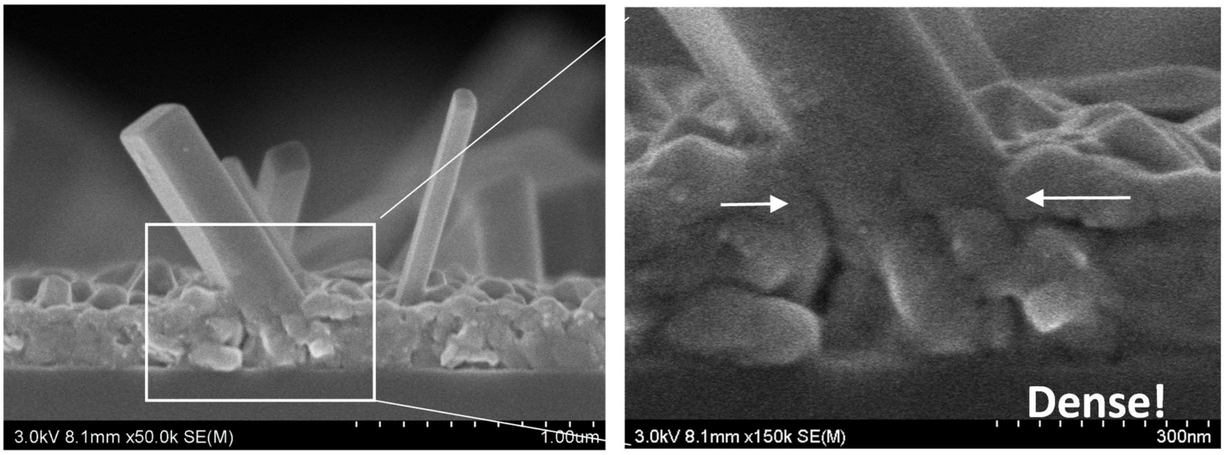

Figure S2. FE-SEM image showing the cross-sectional view of a perovskite nanorod.

The observation suggests that the nanostructure did not grow from a part of the grain, but rather, the entire grain itself acted as the seed.

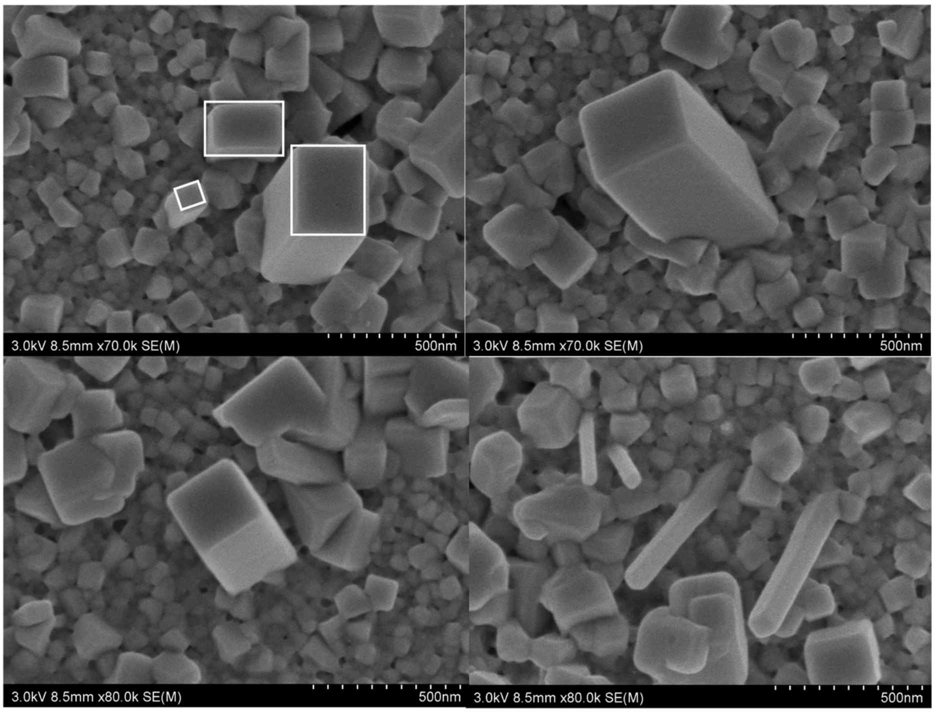

Figure S3. FE-SEM images showing the dimensional variation and aspect ratios of perovskite nanorods.

The cross-sectional dimensions and aspect ratios differ from rod to rod. The growth direction and initial cross-sectional size may depend on the local environment during seed formation.

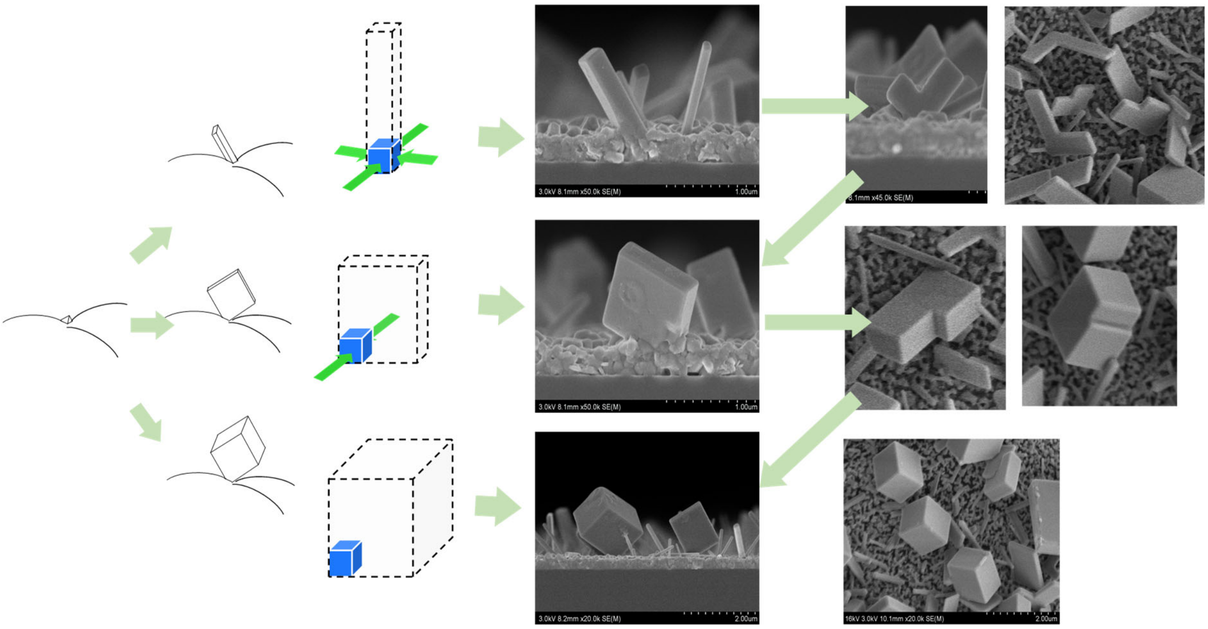

Figure S4. FE-SEM images of nanorods, plates, and cuboids exhibiting unique transitional shapes, such as rod-to-plate and plate-to-cuboid transformations.

These observations suggest that the compressive stress within the seed can be gradually released through the dissolution of surrounding pre-positioned grains. In other words, the shape of the nanostructure might be modulated by controlling the compressive stress,
